# A Split-Path Schema-Based RFID Data Storage Model in Supply Chain Management

**DOI:** 10.3390/s130505757

**Published:** 2013-05-03

**Authors:** Hua Fan, Quanyuan Wu, Yisong Lin, Jianfeng Zhang

**Affiliations:** 1 School of Computer Science, National University of Defense Technology, Changsha 410073, China; E-Mails: quanyuan.wu@gmail.com (Q.W.); jfzhang@nudt.edu.cn (J.Z.); 2 PLA General Logistics Department, Logistics Science Research Institute, Beijing 100071, China; E-Mail: linyisong@live.cn

**Keywords:** RFID technology, data storage model, data compression, data processing, split-path schema, supply chain management

## Abstract

In modern supply chain management systems, Radio Frequency IDentification (RFID) technology has become an indispensable sensor technology and massive RFID data sets are expected to become commonplace. More and more space and time are needed to store and process such huge amounts of RFID data, and there is an increasing realization that the existing approaches cannot satisfy the requirements of RFID data management. In this paper, we present a split-path schema-based RFID data storage model. With a data separation mechanism, the massive RFID data produced in supply chain management systems can be stored and processed more efficiently. Then a tree structure-based path splitting approach is proposed to intelligently and automatically split the movement paths of products. Furthermore, based on the proposed new storage model, we design the relational schema to store the path information and time information of tags, and some typical query templates and SQL statements are defined. Finally, we conduct various experiments to measure the effect and performance of our model and demonstrate that it performs significantly better than the baseline approach in both the data expression and path-oriented RFID data query performance.

## 1. Introduction

Radio Frequency IDentification (RFID) is a kind of automatic identifying technology that allows objects, places or persons to be automatically identified at a distance without a direct line-of-sight, using an electromagnetic challenge/response exchange [1,2]. RFID has played a significant role in minimizing process costs for firms with a high value information service. With the development of low-cost passive RFID tags and vigorous RFID standardization efforts, RFID technology has become an indispensable technology in modern supply chain management [1]. The use of RFID in the supply chain management process has contributed a lot to the aspects of accuracy, information visibility and improved customer service, and supported various cost reduction factors ranging from inventory management to information and labor cost [3]. Although RFID system can provide plenty of data essential to controlling and understanding business processes, applications like supply chain management or real-time tracking may generate such a huge volume of information that it cannot be handled by traditional approaches [4]. More and more space and time are needed to store and process such huge amounts of RFID data. For example, it is predicted that only one company, such as WalMart, will generate over 7 terabytes of operational RFID data per day if it operates RFID on the item level [5]. Therefore, the storage and processing of RFID data is therefore widely considered as a principal challenge and has been an important research topic [6,7].

How can companies store and process the enormous volume of data that an RFID application will generate is a great challenge. In this paper, we present a split-path based RFID data storage model. We found that the database will have mass redundant data if we store the path of each tag independently. Therefore, the corresponding solution is proposed for the purpose of reducing the redundancy in RFID database. All the whole paths of tags have been split into two sections, and then a cluster analysis for the tags with the same path section information, including the locations and time, will be done. We also propose the corresponding approach and algorithm for splitting the whole path. Further, we design a new relational schema to store the path information and the time information for tags. The contributions of this study are as follows:

(1) Split-path based RFID data storage model. There is data redundancy in the RFID technology based supply chain management system, and there are a lot of tags with different information for the whole path but the same path information for some path sections. In the proposed model, the whole path will be split into two sections, and then be stored separately to reduce the system data redundancy.
(2) Tree structure based path splitting approach. In the supply chain, products usually have two processes successively, concentration and distribution. For this reason, there will be some positions with very high in-degree and out-degree in the supply chain. We propose a tree structure based path splitting approach, and the whole paths can be split intelligently and automatically. The large product distribution center is often the root node of the tree structure.
(3) New relational schema for RFID data storage. Based on the proposed new storage model, we redesign the relational schema to store the path information and time information of tags.
(4) Query translation. For the changes of storage model and relational schema, the original query templates have to change accordingly. Some new typical query templates are defined, and we also devise the corresponding SQL statements of these queries.

The rest of this paper is organized as follows: we discuss the related work in Section 2. Section 3 introduces the split-path based RFID data storage model and the tree structure based path splitting algorithm. In Section 4, we describe the new relational schema for RFID data storage in an RDBMS. An empirical evaluation of our solution is reported in Section 5 while our conclusions are presented in Section 6.

## 2. Related Work

With the development of RFID technology, more and more research on RFID data management has been done recently, such as RFID data warehousing and duplicate elimination [8–10], RFID data querying [7,11–13], RFID data cleaning [14–16], and so on. In this section, we will review the existing RFID data compression and processing approaches that are related to our work.

The special way in which RFID device gets data brings more redundancy to the RFID data sets. In recent years, several efforts have been made in the related research field. Mahdin *et al.* [8] proposed a data filtering approach that efficiently detects and removes duplicate readings from RFID data streams. However, the approach has its limitation that all filtering process is aimed at the raw data at reader level rather than the path records of tags. Gonzalez *et al.* [9] proposed a movement graph model as a compact representation of RFID data sets. It provides a clean and concise representation of large RFID data sets. This approach is based on the assumption that the products tend to move and stay together and thus can be grouped together based on their locations. However, it is useless for warehousing of products with scattered movements, and the path oriented queries is inefficient. Bashir *et al.* [10] propose an energy-efficient in-network RFID data filtering scheme to filter duplicate readings in wireless sensor network. In this schema, a clustering mechanism is used to eliminate the duplicate data, and cluster heads only need to forward filtered data towards the base station.

Bai *et al.* [11] proposed a stream query language to provide comprehensive temporal event detection, through temporal operators and extension of sliding-window constructs, and it can support the general RFID data processing for a large variety of RFID applications. Park *et al.* [12] proposed an effective technique for indexing RFID continuous queries. This technique can convert a number of segments into compressed data and store the result as one object. Furthermore, a transform technique is proposed to find a repeated group of segments and convert the group into compressed data. Wilfred *et al.* [13] presented a holistic framework that supports data querying and analysis of raw datasets obtained from different RFID collection points managed by supply chains. Lee *et al.* [7] proposed a path encoding schema to process a massive amount of RFID data for supply chain management. By using two numbers, the paths that satisfy the conditions can be found easily. However, with the increasing of the tag numbers in system, the storage cost of data and the time cost of data query will increase rapidly.

Our work on the RFID data compression and processing makes use of several traditional data processing techniques. In this paper, we propose a split-path based RFID data storage model that improves on the storage model used in Reference [7] to reduce the storage cost and speed up query processing.

## 3. Split-Path Based RFID Data Storage Model

In the RFID technology based supply chain management system, when the product with RFID tag moves through the detection region of the reader, it will be detected by the reader and a record will be generated in the form of (*tag_id*, *reader_id*, *timestamp*), where *tag_id* and *reader_id* refer to EPCs which universally uniquely identify the tagged item and the RFID reader (readers are usually fixed at a specific location, so *reader_id* and the locations *LOC* in supply chain are in one-to-one correspondence), and the *timestamp* is the time when the reading occurred [17]. In the work of Reference [7], the raw RFID data generated in supply chain management have been translated into the form of (*tag_id*, *loc*, *start_time*, *end_time*), which is a set of stay records and has no duplicates. *loc* is the location of the RFID reader which detects the tag; *start_time* and *end_time* are the time when the tag enters and leaves the location, respectively. Furthermore, path records are constructed to instead of raw RFID data in the form of *L*_1_[*s*_1_, *e*_1_]→…→*L_i_*[*s_i_*, *e_i_*]→…→*L_n_*[*s_n_*, *e_n_*], where *L_i_* is the location where the tag is detected, *s_i_* and *e_i_* are the *start_time* and *end_time* at the location *L_i_*, respectively.

**Figure 1.**
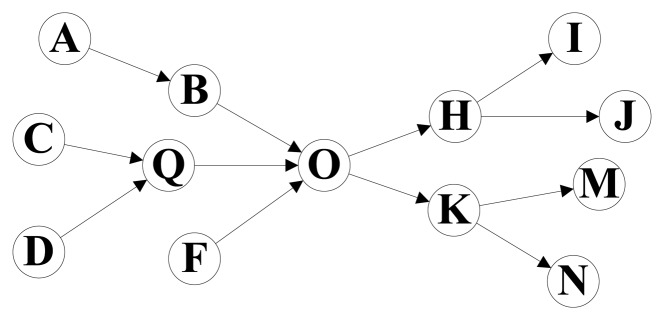
Path graph of an electronics supply chain.

Figure 1 shows the path graph of an electronics supply chain. The node A, C, D and F mean several manufacturers, and node I, J, M and N are the electronics retailers. Other nodes mean middlemen, and node O is the biggest electronics distributing center in this supply chain. The path information of products in this supply chain for a period is stored in Table 1. We found that the storage cost can be significantly reduced if we split the whole path of each product into two sections by the distributing center node O and store the information of each path section separately, and the performance of path oriented RFID data queries can also be improved. All the path records in Table 1 can be expressed by the combination of two path sections in Table 2. For example, the path information of Tag 1 can be represented by the path sections So_4 and Si_2, and all the 18 long path records in Table 1 can be represented by the 10 short path section records. The whole path graph is separated into two tree structures by the center node, and the tree structures represent the concentration and distribution of products, respectively. The topology similar to Figure 1 is very common in the RFID technology based applications, such as supply chain management, logistics management, and so on.

### 3.1. Tree Structure Based Path Splitting Algorithm

In the last section, we found that we can reduce the storage cost and improve the performance of path oriented RFID data queries by splitting the whole path of each product into two sections and storing the information of each path section in database separately. Therefore, we proposed a tree structure based path splitting algorithm in this section. First, the definitions of several important concepts are given as below:

Definition 1 Path graph *G*(*V*, *E*) is a directed acyclic graph representing the moving path of tags. *V* is the set of locations, *E* is the set of transitions between locations. An edge *e* = (*v_i_*, *v_j_*) indicates that tags can move from location *v_i_* to location *v_j_*.
Definition 2 The **successor node set** of node *v* is the set of all the successor nodes of node *v* in *G*, denoted as *Suc*(*v*), while the **precursor node set** of node *v* is the set of all the precursor nodes of node *v* in *G*, denoted as *Pre*(*v*).
Definition 3 A **whole path*** p* is a full movement path from a source node to a sink node in *G* in the form of *v*_0_→*v*_1_→…→*v_n_*, where *v*_0_ is the source node and *v_n_* is the sink node in *G*. The node set and edge set of the path *p* are denoted as *C_V_*(*p*) and *C_E_*(*p*). The whole path set, *P*, is the set of all the whole paths in *G*. Unless specifically mentioned, the word “path” means whole path in this paper.
Definition 4 If a whole path *p* in *G* is split into two sections by a node *v_i_* ∈ *C_V_*(*p*), then the section before *v_i_* is called the **source section** of *p* and the section after *v_i_* is called the **sink section** of *p*. We denote the set of source sections which end with the node *v_i_* as $S_{v_{i}}^{b}$, and $S_{v_{i}}^{f}$ is the set of sink sections which start with the node *v_i_*. In particular, $S_{v_{i}}^{b}=\{{\tilde{v}}_{i}\}$ when the node *v_i_* is a source node in *G*, and $S_{v_{i}}^{f}=\{{\tilde{v}}_{i}\}$ when the node *v_i_* is a sink node in *G*, where *ṽ_i_* is a special path composed of only one node. For example, in Figure 1, $S_{O}^{b}=\{\text{A}\to \text{B}\to \text{O},\text{C}\to \text{Q}\to \text{O},\text{D}\to \text{Q}\to \text{O},\text{F}\to \text{O}\}$, $S_{O}^{f}=\{\text{O}\to \text{H}\to \text{I},\text{O}\to \text{H}\to \text{J},\text{O}\to \text{K}\to \text{M},\text{O}\to \text{K}\to \text{N}\}$, $S_{A}^{b}=\{\tilde{A}\}$ and $S_{I}^{f}=\{\tilde{I}\}$.
Definition 5 For any node *v* ∈ *V* (or edge *e* ∈ *E*), if there exists a path *p* ∈ *P* that satisfy *v* ∈ *C_V_*(*p*) (or *e* ∈ *C_E_*(*p*)), then we can say that *p* is the **covered path** of *v* (or *e*) in *G*. The **covered path set** of node *v*, *U_P_*(*v*), is defined as:
  (1) $$U_{P}(\nu )=\{p\in P\;|\nu \in C_{V}(p)\}$$
Definition 6 Given *v* ∈ *V*, for any node *v_i_* ∈ *V*, if there exists a path *p* ∈ *P* that satisfy *v_i_* ∈ *C_V_*(*p*) and *v* ∈ *C_V_*(*p*), then we can say that *v_i_* is the **covered node** of *v* in *G*. The **covered node set** of node *v*, *U_N_*(*v*), is defined as:
  (2) $$U_{N}(\nu )=\{\nu_{i}\in C_{V}(p)\;|\;p\in U_{P}(\nu )\}$$
Definition 7 Given *v* ∈ *V*, for any edge *e_i_* ∈ *E*, if there exists a path *p* ∈ *P* that satisfy *e_i_* ∈ *C_E_*(*p*) and *v* ∈ *C_V_*(*p*), then we can say that *e_i_* is the **covered edge** of *v* in *G*. The **covered edge set** of node *v*, *U_E_*(*v*), is defined as:
  (3) $$U_{E}(\nu )=\{e_{i}\in C_{E}(p)\;|\;p\in U_{P}(\nu )\}$$
Definition 8 For any node *v_i_* ∈ *U_N_*(*v*), if there does not exist a path *p* ∈ *P* that satisfies *v_i_* ∈ *C_V_*(*p*) and *v* ∉ *C_V_*(*p*), then we can say that *v_i_* is the **full-covered node** of *v* in *G*. The **full-covered node set** of node *v*, *Uˆ_N_*(*v*), is defined as:
  (4) $${\hat{U}}_{N}(\nu )=\{\nu_{i}\in U_{N}(\nu )\;|\;U_{P}(\nu_{i})\subseteq U_{P}(\nu )\}$$
Definition 9 For any edge *e_i_* ∈ *U_E_*(*v*), if there does not exist a path *p* ∈ *P* that satisfies *e_i_* ∈ *C_E_*(*p*) and *v* ∉ *C_V_*(*p*), then we can say that *v_i_* is the **full-covered edge** of *v* in *G*. The **full-covered edge set** of node *v*, *Uˆ_E_*(*v*), is defined as:
  (5) $${\hat{U}}_{E}(\nu )=\{e_{i}\in U_{E}(\nu )\;|\;U_{P}(e_{i})\subseteq U_{P}(\nu )\}$$

The path splitting method will influence the effect of data compression directly, but splitting paths in a path graph optimally is an NP-hard problem. Therefore, we propose a heuristic path splitting approach called tree structure based path splitting algorithm. The implementation of the proposed approach is an iterative process, and the main procedure consists of nine steps as described below:

Step 1 Compute the *section-tuples*. Compute the 4-tuples { $d_{v}^{b}$, $l_{v}^{b}$, $d_{v}^{f}$, $l_{v}^{f}$} of each node *v* in *G*, where $d_{v}^{b}$ and $d_{v}^{f}$ are the size of the sets $S_{v}^{b}$ and $S_{v}^{f}$, and $l_{v}^{b}$ and $l_{v}^{f}$ are the average length of the path sections in $S_{v}^{b}$ and $S_{v}^{f}$, respectively. We present a simple method to compute the section-tuples of each node, and there is an iterative procedure for the computation of each element in the section-tuples.
  (6) $$d_{v}^{b}=\{\begin{array}{cc} 1 & \text{if}\;\text{Pre}\;(v)=\emptyset \\ \sum_{v\prime \in \;\text{Pre}(v)}d_{v\prime }^{b} & \text{otherwise} \end{array}$$
  (7) $$d_{v}^{f}=\{\begin{array}{cc} 1 & \text{if}\;\text{Suc}\;(v)=\emptyset \\ \sum_{v\prime \in \;\text{Suc}(v)}d_{v\prime }^{f} & \text{otherwise} \end{array}$$
  (8) $$l_{v}^{b}=\{\begin{array}{cc} 0 & \text{if}\;\text{Pre}(v)=\emptyset \\ \frac{\sum_{v\prime \in \;\text{Pre}(v)}l_{v\prime }^{b}d_{v\prime }^{b}}{\sum_{v\prime \in \;\text{Pre}(v)}l_{v\prime }^{b}}+1 & \text{otherwise} \end{array}$$
  (9) $$l_{v}^{f}=\{\begin{array}{cc} 0 & \text{if}\;\text{Suc}(v)=\emptyset \\ \frac{\sum_{v\prime \in \;\text{Suc}(v)}l_{v\prime }^{f}d_{v\prime }^{f}}{\sum_{v\prime \in \;\text{Suc}(v)}d_{v\prime }^{f}}+1 & \text{otherwise} \end{array}$$
Step 2 Compute the *covered-path* number. Compute the *covered-path* number of each node *v* (*i.e.*, the size of the *covered path set* of *v*, |*U_P_*(*v*)|) which defines its *path-degree*, *d_v_*, as:
  (10) $$d_{v}=\left|U_{P}(v)\;\right|=d_{v}^{b}d_{v}^{f}$$
  *Proof:* |*U_P_*(*v*)| is the size of the covered path set of *v*. Let *Y_v_* be the Cartesian product of $S_{v}^{b}$ and $S_{v}^{f}$. Here, the Cartesian product is the set of the whole paths connected by any two path sections respectively from $S_{v}^{b}$ and $S_{v}^{f}$ via the common node *v*. $d_{v}^{b}$ and $d_{v}^{f}$ are the size of $S_{v}^{b}$ and $S_{v}^{f}$ respectively, thus $d_{v}^{b}d_{v}^{f}$ is the size of *Y_v_*. For any path *p* ∈ *U_P_*(*v*), we can get *v* ∈ *C_V_*(*p*) by an application of Definition 5. The path *p* can be split into two sections by the node *v*, the source section *p*_1_ and the sink section *p*_2_. Applying Definition 4 we have $p_{1}\in S_{v}^{b}$ and $p_{2}\in S_{v}^{f}$, then *p* ∈ *Y_v_* holds. Hence, $\left|U_{P}(v)\right|\leq d_{v}^{b}d_{v}^{f}$. Conversely, for any *p*′ ∈ *Y_v_*, combined by two path sections from $S_{v}^{b}$and $S_{v}^{f}$ (*v* ∈ *C_V_*(*p*′)) respectively, which is a path in *G* (*p*′ ∈ *P*) obviously, we can get *p*′ ∈ *U_P_*(*v*) by applying Definition 5. Hence, $d_{v}^{b}d_{v}^{f}\leq \left|U_{P}(v)\right|$. Combining the two inequalities above, we see that it suffices to require that $\left|U_{P}(v)\right|=d_{v}^{b}d_{v}^{f}$. This completes the proof.
Step 3 Compute the *length-difference*. Each node can split all its *covered-paths* into two sections, source sections and sink sections. The *length-difference* Δ*_v_* of each node can be computed by
  (11) $$\Delta_{v}=\left|l_{v}^{f}-l_{v}^{b}\;\right|$$
Step 4 Compute the *throughput ratio*. The throughput of a specific node means the number of tags which have been in the node. Therefore, we can compute the throughput ratio, *H_v_*, by the throughput of the current node to the sum of tags in the whole system as:
  (12) $$H_{v}=\frac{D_{v}}{\sum_{T_{i}}\left\{\exists t,\exists n\in V,\;\text{location}{\left(T_{i}\right)}_{t}=n\right\}}$$
  (13) $$D_{v}=\sum_{T_{i}}\left\{\exists t,\text{location}{\;(T_{i})}_{t}=v\right\}$$
  where, *location*(*T_i_*)*_t_* is the location of tag *T_i_* at time *t*, and *D_v_* is the throughput of node *v*.
Step 5 Calculate the combined weight. Calculate the combined weight *W_v_* for each node *v*, as
  (14) $$W_{v}=w_{d}d_{v}-w_{\Delta }\;\Delta_{v}+w_{H}H_{v}$$
  where, *w_d_*, *w*_Δ_ and *w_H_* are the weighting factors for the corresponding system parameters. Note that these weighting factors can be chosen as needed such that *w_d_*+*w*_Δ_+*w_H_* =1. The contribution of the individual components can be tuned by choosing the appropriate combination of the weighting factors. The first two components in the Equation (14), *w_d_* and *w*_Δ_, are directly related to the topology of the path graph, while the third component *w_H_* is directly related to the real movement distribution of tags in a period. Therefore, we can get a common path splitting schema based on the topology of the path graph by increasing the values of *w_d_* and *w*_Δ_. In contrast, if we want to get the path splitting schema which can preferably apply to the storage of the information of the existing tags in system, we should increase the value of *w_H_*.
Step 6 Choose the *root-node*. Choose the node with the biggest *W_v_* as the *root-node*, denoted as *R_i_*.
Step 7 Construct the *path trees*. With the root of *R_i_*, construct two tree structures, the forward tree *T_if_* and the backward tree *T_ib_*. The construction of *T_if_*: First, for each out-edge of *R_i_* in *G*, add a new branch and a corresponding child node in *T_if_*; Then, do the same process to all its child nodes and grandchild nodes in *T_if_* until all the leaf nodes in *T_if_* do not have out-edges in *G*. Likewise, the backward tree, *T_ib_*, can be constructed by doing the similar processes to *R_i_* and the in-edges in *G*. After that, any path *p* ∈ *U_P_*(*R_i_*) can be represented together by the path sections in *T_if_* and *T_ib_.*
Step 8 Remove the *full-covered sets*. Remove the nodes in *Uˆ_N_*(*R_i_*) and the edges in *Uˆ_E_*(*R_i_*) from *G*, for the covered path sets of these nodes and edges have been included in the trees *T_if_* and *T_ib_*. Therefore, *V* = *V* − *Uˆ_N_(R_i_)* and *E* = *E* − *Uˆ_E_(R_i_)*.
Step 9 Repeat Steps 1–8 for the remaining nodes in *G* until *V* = ø.

After processing by our splitting algorithm, the path graph of a complex supply chain will be split into two groups of trees, the forward trees and the backward trees. The forward tree (*T_if_*) represents the path information of the tag after it reaches the root node, and its direction is from the root node to leaf nodes; the backward tree (*T_ib_*) represents the path information of the tag before it reaches the root node, and its direction is from leaf nodes to the root node. All the leaf nodes in forward trees are the sink nodes in *G*, and all the leaf nodes in backward trees are the source nodes in *G*. Therefore, the path information of each tag will be split into two sections and stored into the two different trees separately.

### 3.2. An Illustrative Example of Path Splitting

**Figure 2.**
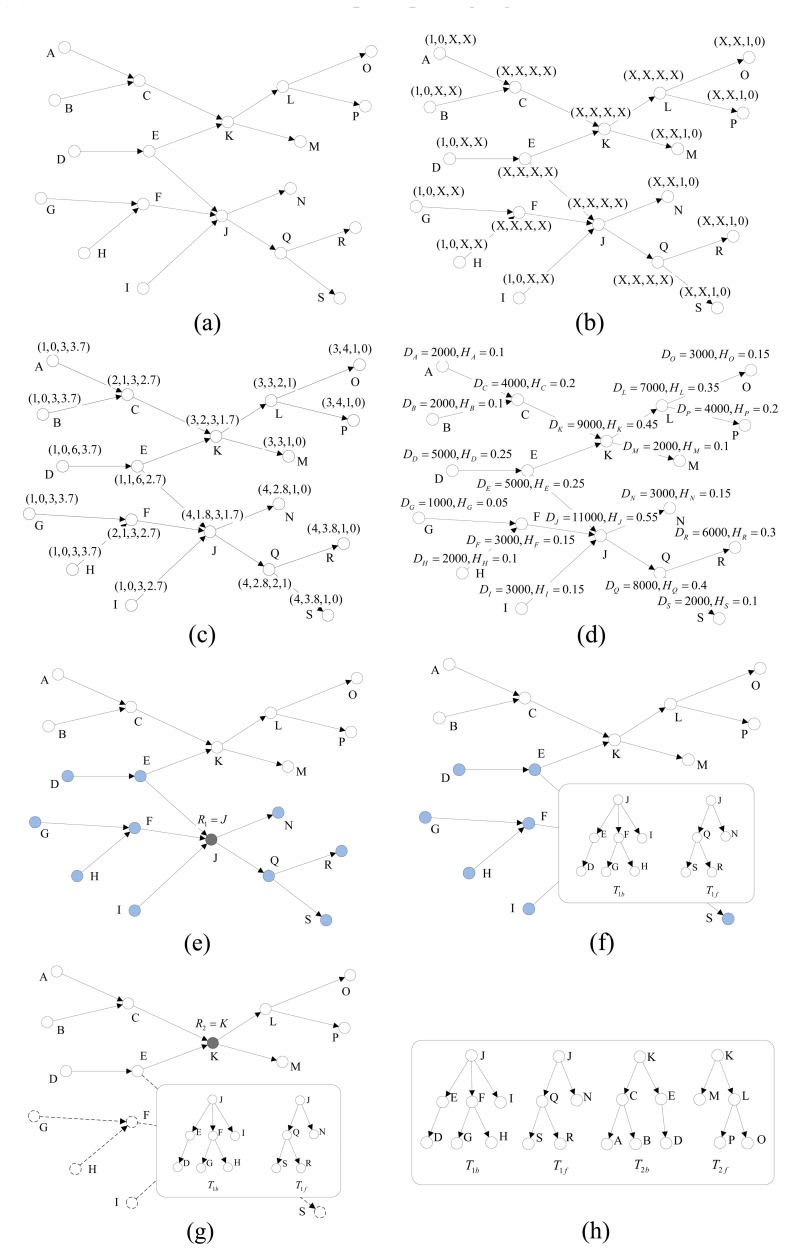
An illustrative example of path splitting. (**a**) Topologic structure of the supply chain; (**b**) Recursive process of the computation of the section-tuples; (**c**) Section-tuples of the nodes; (**d**) Throughput and throughput ratio of nodes; (**e**) The root-node and its covered node set; (**f**) The construction of forward tree and backward tree; (**g**) Remove full-covered set. (**h**) The result of tree structure based path splitting algorithm.

We demonstrate our algorithm with the help of Figure 2. All the numeric values obtained from executing the path splitting procedure on the 19 nodes in Figure 2(a), are tabulated in Table 3. nto two sections, “A→C→K” and “K→L→O”, and its root-node is K.

Figure 2(a) shows all the locations and paths in the path graph. The lettered nodes represent the different locations, and the directed edges between nodes signify that tags can move in the direction of the arrow. Figure 2(b,c) show the recursive process of the computation of the section-tuples in Step 1, and we use the symbol “X” to represent the values that have not be calculated yet in Figure 2(b).

The path-degree, *d_v_*, of each node is computed in Step 2, and the length-difference, Δ*_υ_*, of each node is calculated as Step 3. As shown in Figure 2(d), based on the value of *D_v_* we can calculate the throughput ratio, *H_v_*, of each node. After the values of all the components are identified, we compute the weighted metric, *W_v_*, of each node as proposed in Step 5 of our algorithm. Here, the weighting factors considered are ***w****_d_* = 0.4, ***w***_Δ_ = 0.1 and ***w****_H_* = 0.5, which is a relatively balanced choice. Figure 2(e) shows how a node with maximum *W_v_* is selected as the root-node as stated in Step 6 of our algorithm. The solid black node represents the root-node elected for the path graph, and the blue crosshatched nodes represent the covered node set of the root-node. Figure 2(f) shows the forward tree and backward tree constructed by execution of the Step 7. As shown in Figure 2(g), the nodes in the full-covered node set and the edges in the full-covered edge set of root-node have been removed, and the solid black node is another root-node elected in the next election procedure. Figure 2(h) shows the final result, all the forward trees and backward trees constructed by our algorithm. Therefore, each whole path can be represented by two path sections, and the source section is stored in a backward tree while the sink section is stored in a forward tree. The two trees have a collective root-node which is also called the split-node of this whole path. It can be easily proved that there must be one and only one split-node for each whole path. As shown in Figure 2(h), the whole path “A→C→K→L→O” have been split into two sections, “A→C→K” and “K→L→O”, and its root-node is K.

## 4. Relational Schema for RFID Data Storage in a RDMBS

The main aim of splitting paths is to effectively reduce the storage cost of path information and improve the performance of path oriented data queries, but which also requires us to do the corresponding adjustment to the data storage scheme and the way of data query.

**Figure 3.**
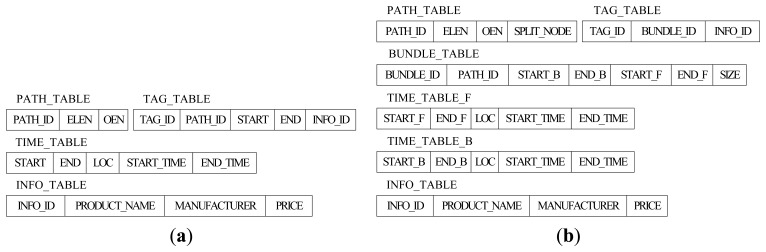
(**a**) Original relational schema; (**b**) New relational schema to store RFID data.

Figure 3(a) shows the original relational schema to store RFID data in Reference [7]. The size of TAG_TABLE is related to the number of tags, so it is impossible to reduce the size of TAG_TABLE. However, there are many tags moving and staying together through the whole path, so we can reduce the storage cost by representing such a collective movement by a single record no matter how many tags were originally collected. For this reason, BUNDLE_TABLE has been added in our relational schema. There are seven fields in BUNDLE_TABLE. *PATH_ID* is the identifier for the path information and (*START_B*, *END_B*) and (*START_F*, *END_F*) are the identifiers for the time information of the corresponding source section and sink section. In addition, *SIZE* is the tag number of current bundle. The new TAG_TABLE in Figure 3(b) only has three fields, *TAG_ID*, *BUNDLE_ID* (the identifier for BUNDLE_TABLE) and *INFO_ID* (the identifier for INFO_TABLE).

Obviously, the size of PATH_TABLE is related to the number of paths in the path graph, and it will be reduced if we store the split path sections instead of the whole paths. However, compared with TIME_TABLE and TAG_TABLE, the size of PATH_TABLE is much smaller. Therefore, reducing the size of PATH_TABLE has little effect on reducing the whole storage cost, and the efficiency of path oriented queries might be influenced by the complex structure of split PATH_TABLE, instead. For this reason, the structure of PATH_TABLE remains the same as before except an added label of *SPLIT_NODE*. A more detailed overview of the path encoding schema can be found in Reference [7], which we will introduce briefly in this paper. Each location in the path graph is associated with a different prime number, and the prime number for location *L_a_* is denoted by *Prime*(*L_a_*). The Element List Encoding Number of the path *p_i_*: *L*_1_→*L*_2_→…→*L_n_* is given by *ELEN*(*p_i_*) = *Prime*(*L*_1_) × *Prime*(*L*_2_) × … × *Prime*(*L_n_*), and we can get the locations that compose *p_i_* by the Element List Encoding Number *ELEN*(*p_i_*) based on the Fundamental Theorem of Arithmetic. The path *p_i_* contains location *L_a_* if and only if *ELEN*(*p_i_*) mod *Prime*(*L_a_*) = 0. The Order Encoding Number of *p_i_*, *OEN*(*p_i_*), is the number with 0 ≤ *OEN*(*p_i_*) ≤ *ELEN*(*p_i_*) computed by the Chinese Remainder Theorem, and we can know the order information for any location *L_a_* in the path by computing *OEN*(*p_i_*) mod *Prime*(*L_a_*) [7]. Suppose that the locations, *L_a_* and *L_b_*, are the locations in the same path *p_i_*, they have the parent-child relationship (*i.e.*, *L_a_*/*L_b_*) if and only if *OEN*(*p_i_*) mod *Prime*(*L_a_*) < *OEN*(*p_i_*) mod *Prime*(*L_b_*) holds. Likewise, *L_a_* and *L_b_* have the ancestor-descendant relationship (*i.e.*, *L_a_*//*L_b_*) if and only if *OEN*(*p_i_*) mod *Prime*(*L_a_*) + 1 = *OEN*(*p_i_*) mod *Prime*(*L_b_*). The symbols, “/” and “//”, are used to represent the parent-child and ancestor-descendant relationship between locations in this paper, respectively.

**Figure 4.**
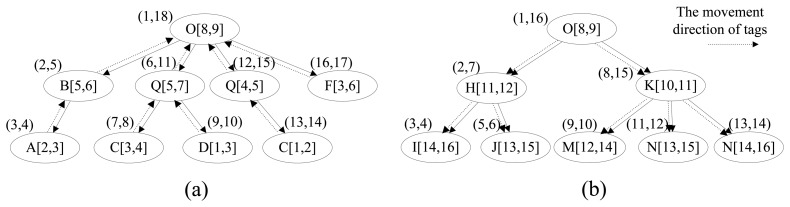
Time tree. (**a**) The backward time tree; (**b**) The forward time tree.

Except for TAG_TABLE, TIME_TABLE is the biggest table, and its size will unceasingly increase over time. Hence, reducing the size of TIME_TABLE is the key point to reduce the storage cost, and it is also one of the main emphases of this paper. Based on the forward trees and backward trees constructed in the last section, we construct the time trees to store the time information for the tags in which the node has the start time and end time as well as the location. Similarly, there are also two types of time tree, forward time tree and backward time tree. The forward time tree is responsible for storing the time information corresponding to the sink sections, and the backward time tree is responsible for storing the time information correspond to the source sections. The different nodes in the time tree represent different paths even though they have the same node id, such as C[3,4] and C[1,2] in Figure 4(a). The region-based numbering schema [18,19], which assigns a node with two values (*START_F* and *END_F* in forward time tree, or *START_B* and *END_B* in backward time tree), is used in the time tree. It encodes the starting and ending positions of a node in a path to identify the node so that the ancestor/descendant relationship between two nodes can be determined by merely examining their codes. Such a numbering schema can greatly improve the path oriented data query performance. *START_F* (*START_B*) and *END_F* (*END_B*) are assigned consecutively during the depth-first search. In the forward time tree, the region numbering has the property that node A is the ancestor of node B (A is also the precursor of B in the path) if and only if *A.START_F* < *B.START_F* and *B.END_F* < *A.END_F*. By contrast, in the backward time tree, the region numbering has the property that node A is the ancestor of node B (actually, A is the successor of B in the path) if and only if *A.START_B* < *B.START_B* and *B.END_B* < *A.END_B*.

In our relational schema, there are two time tables, TIME_TABLE_B and TIME_TABLE_F, corresponding to the backward time trees and forward time trees, respectively. The split-node (root-node) of a whole path will present in both corresponding forward time tree and backward time tree. To retrieve the time information conveniently and efficiently, we assign the region numbers that correspond to the source node (*START_B* and *END_B*) and sink node (*START_F* and *END_F*) in the path record of the tags in the specific bundle to the bundle. As shown in Figure 4, the time trees are constructed from the path records in Table 2.

We can get the time and location information for tags and bundles by their region numbers of source node and sink node. For example, if we know the region numbers of the source node and sink node of tag 11 are (7,8) and (11,12), respectively, we can retrieve the nodes satisfying *START_F* ≤ 7 and *END_F* ≥ 8 in forward time tree and *START_B* ≤ 11 and *END_B* ≥ 12 in backward time tree, such as C[3, 4], Q[5, 7], O[8, 9], K[10, 11] and N[13, 15]. We have not changed the INFO_TABLE in which the information of products such as manufacturer, price and name are stored, and the whole structure of our relation schema is shown in Figure 2(b). We take the data in Tables 1 and 2 for example to compare the changes of data storage based two different schemas. Limited by the length of paper, we only show the details of TIME_TABLE in original schema and TIME_TABLE_B and TIME_TABLE_F in our new schema. As shown in Table 4, the introduction of the split-path based RFID data storage model has greatly improved the storage efficiency and made the time information records in TIME_TABLE reduce from 41 to 17.

The split-path based storage model not only can reduce the storage overhead, but also can improve the path oriented data query performances. First, the smaller sizes of the TIME_TABLE_B and TIME_TABLE_F can reduce the time cost of scanning the time information table. In addition, the adoption of the BUNDLE_TABLE enables many queries not to have to scan the TAG_TABLE, so that the execution time of queries can be reduced further.

## 5. Experimental Evaluation

In this section, we report our comprehensive evaluation of the proposed model and algorithms. All the experiments were conducted on an Intel(R) Core(TM) 2 Duo CPU T9550 @2.66 GHz 2.67 GHz system with 2 GB of RAM, running Windows 7. The RDBMS we used to store RFID data is Microsoft SQL server 2005. In the experiments, we consider a comparative data size and query performance analysis of the path encoding scheme based model [7], denoted as Path, and the proposed model denoted as Split-Path.

The simulation data for our experiments were generated by a synthetic RFID data generator that simulates the operation of RFID readers in supply chain management environment. We suppose that there are 222 different positions in the whole supply chain, including two main concentration and distribution centers, 20 wholesalers, 100 manufacturers and 100 retailers. The average length of the paths is 5. There are six sets of data, which respectively include 1 × 10^6^ tags, 2 × 10^6^ tags, 4 × 10^6^ tags, 6 × 10^6^ tags, 8 × 10^6^ tags and 1 × 10^7^ tags, for testing the performance of our methods in the processing of RFID data with different sizes.

### 5.1. Query Set and Query Translation

As shown in Table 5, 9 representative queries are formulated to test various features of our model. Q1 is a tracking query, Q2–Q4 are path oriented retrieval queries, and Q5–Q9 are path oriented aggregate queries.

In the experiments, we store RFID data in Microsoft SQL Server, and the queries, including tracking queries and path oriented queries, must be translated into SQL queries. Because the improvement of the relational schema for RFID data storage, we have to update the query translation algorithm to get the corresponding SQL statements. We have listed some representative SQL statements in Tables 6,7 and 8, and *pA*, *pB* and *pC* in the tables below respectively denote *Prime*(*A*), *Prime*(*B*) and *Prime*(*C*):

(Q1) <TAG_ID = my_tag_id>
(Q2) <//A//B/C>
(Q3) <//A//B[(EndTime-StartTime) < 50]/C>

Limited by the length of paper, the rest SQL statements are not listed here.

### 5.2. Data Compression

**Figure 5.**
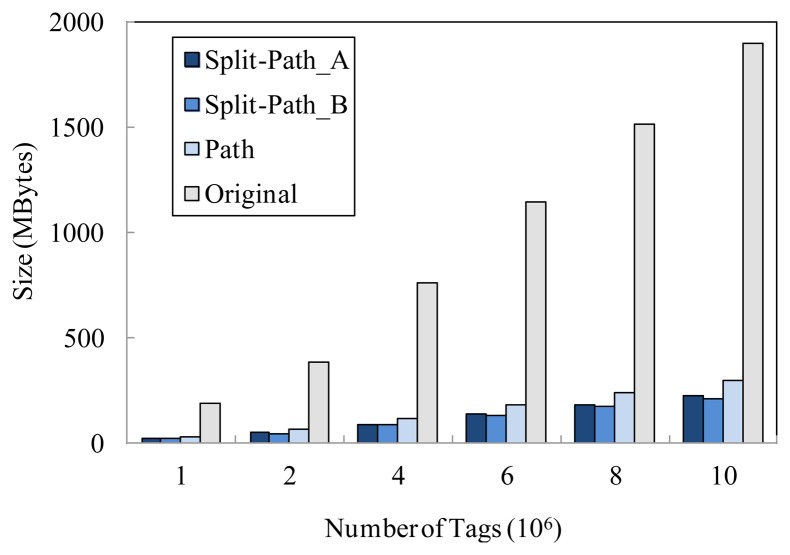
Storage cost comparison under different storage schema.

In Microsoft SQL server, the data is stored in an mdf file. As shown in Figure 5, we compare the storage cost of the proposed model denoted as Split-Path_A(*w_d_* = 0.4, *w*_Δ_ = 0.1 and *w_H_* = 0.5) and Split-Path_B(*w_d_* = 0.3, *w*_Δ_ = 0.05 and *w_H_* = 0.65), the path encoding schema based storage model denoted as Path, and the original raw RFID data denoted as Original. In this experiment, we can clearly see that the storage cost of Split-Path_A and Split-Path_B are always smaller than that of Path. As a matter of fact, the time information storage cost of our model (the total size of TIME_TABLE_B and TIME_TABLE_F) is only 4% of that of the path encoding schema based storage model (the size of TIME_TABLE). It is worthwhile to note that the proposed model can achieve higher compression ratio with the increasing of the tag number. Therefore, the larger the original data size of the system is, the better the effects of data expression is. In addition, we can see that the proposed model with higher value of *w_H_* can preferably apply to the storage of the information of the existing tags in system.

### 5.3. Query Processing

**Figure 6.**
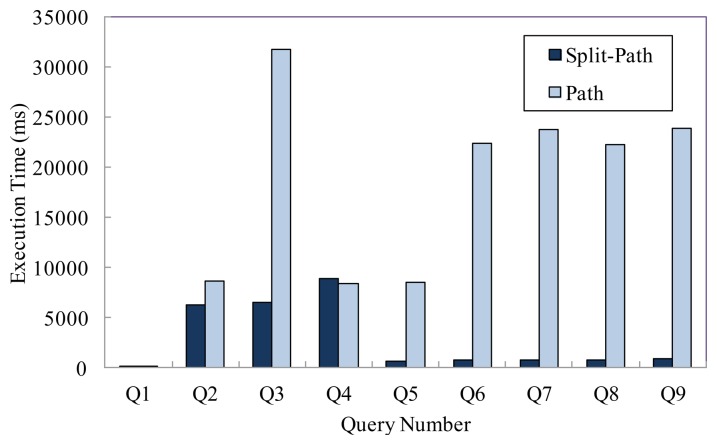
Query performance comparison.

We conduct experimental evaluations for the nine representative queries in Table 5 to validate our approach in this section. In this experiment, the number of tag is 1 × 10^7^, and we compare the query performance of two models under this condition. Figure 6 presents the average execution time of various queries. We can see that the split-path based storage model can achieve better query performance than the path encoding schema based storage model, especially for the path oriented aggregate queries.

**Figure 7.**
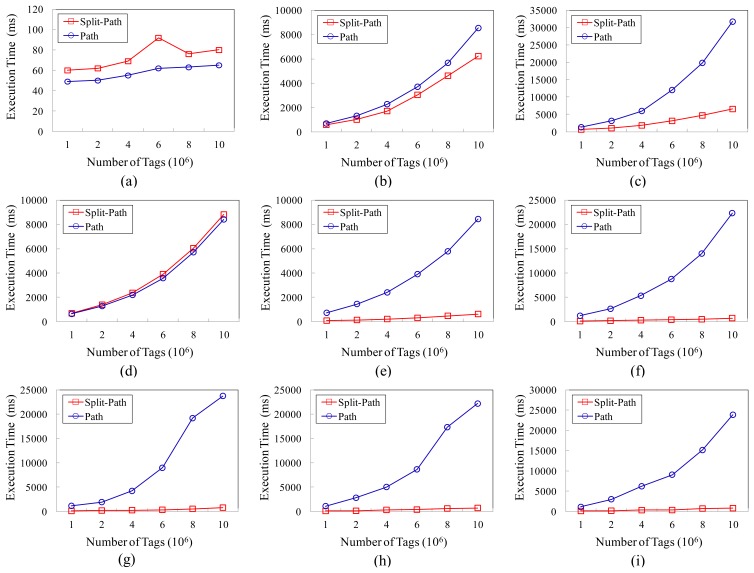
Execution time for representative queries. (**a**) Query 1; (**b**) Query 2; (**c**) Query 3; (**d**) Query 4; (**e**) Query 5; (**f**) Query 6; (**g**) Query 7; (**h**) Query 8; (**i**) Query 9.

We compare the query performance of the two models according to the number of tags, and the results are shown in Figure 7. As shown in Figure 7(a,b,d), the query performances of the two models for Q1, Q2 and Q4 are very close to each other. However, as shown in the rest part of Figure 7, the performances of our approach are obviously better than that of the path encoding schema based storage model, especially for the path oriented aggregate queries. The better query performances of our model benefit from the improvement in two aspects. On the one hand, the smaller sizes of the TIME_TABLE_B and TIME_TABLE_F can reduce the time cost of scanning the time information table; on the other hand, the adoption of the BUNDLE_TABLE enables many queries not to have to scan the TAG_TABLE, so that the execution time have been further reduced.

## 6. Conclusions

In this paper, we present a split-path based RFID data storage model to reduce the time and space overhead of RFID data processing in supply chain management systems. We split all the path records of products into two sections, and the information of these path sections is stored in database separately. Because splitting paths in a supply chain optimally is an NP-hard problem, a heuristic tree structure based path splitting approach is proposed to split the paths intelligently and automatically. In addition, based on the proposed storage model, we design a new relational schema to store the path information and time information of tags, and some typical query templates and the corresponding SQL statements is defined. Finally, the experimental results demonstrate that the proposed model and algorithm provide superior query performance and offer a significant improvement in data compression compared to the baseline approaches.

## Figures and Tables

**Table 1.** The path records of products in supply chain for a period.

**Path Records**
**Tag 1:** A[2, 3] → B[5, 6] → O[8, 9] → H[11, 12] → I[14, 16]
**Tag 2:** C[3, 4] → Q[5, 7] → O[8, 9] → H[11, 12] → J[13, 15]
**Tag 3:** D[1, 3] → Q[5, 7] → O[8, 9] → H[11, 12] → J[13, 15]
**Tag 4:** A[2, 3] → B[5, 6] → O[8, 9] → H[11, 12] → J[13, 15]
**Tag 5:** F[3, 6] → O[8, 9] → H[11, 12] → J[13, 15]
**Tag 6:** C[3, 4] → Q[5, 7] → O[8, 9] → K[10, 11] → M[12, 14]
**Tag 7:** D[1, 3] → Q[5, 7] → O[8, 9] → H[11, 12] → I[14, 16]
**Tag 8:** A[2, 3] → B[5, 6] → O[8, 9] → K[10, 11] → N[13, 15]
**Tag 9:** D[1, 3] → Q[5, 7] → O[8, 9] → K[10, 11] → N[13, 15]
**Tag10:** A[2, 3] → B[5, 6] → O[8, 9] → K[10, 11] → M[12, 14]
**Tag11:** C[3, 4] → Q[5, 7] → O[8, 9] → K[10, 11] → N[13, 15]
**Tag12:** F[3, 6] → O[8, 9] → H[11, 12] → I[14, 16]
**Tag13:** D[1, 3] → Q[5, 7] → O[8, 9] → K[10, 11] → M[12, 14]
**Tag14:** F[3, 6] → O[8, 9] → K[10, 11]→ N[13, 15]
**Tag15:** C[3, 4] → Q[5, 7] → O[8, 9] → H[11, 12] → I[14, 16]
**Tag16:** F[3, 6] → O[8, 9] → K[10, 11] → M[12, 14]
**Tag17:** D[1, 3] → Q[5, 7] → O[8, 9] → K[10, 11] → N[14, 16]
**Tag18:** C[1, 2] → Q[4, 5] → O[8, 9] → H[11, 12] → I[14, 16]

**Table 2.** Path section records.

**Source Sections**	**Sink Sections**
**So_1:** A[2, 3] → B[5, 6] → O[8, 9]	**Si_1:** O[8, 9] → H[11, 12] → I[14, 16]
**So_2:** C[3, 4] → Q[5, 7] → O[8, 9]	**Si_2:** O[8, 9] → H[11, 12] → J[13, 15]
**So_3:** C[1, 2] → Q[4, 5] → O[8, 9]	**Si_3**: O[8, 9] → K[10, 11] → M[12, 14]
**So_4:** D[1, 3] → Q[5, 7] → O[8, 9]	**Si_4**: O[8, 9] → K[10, 11] → N[13, 15]
**So_5:** F[3, 6] → O[8, 9]	**Si_5**: O[8, 9] → K[10, 11] → N[14, 16]

**Table 3.** Execution of path splitting algorithm.

**Node Id**	$d_{v}^{f}$	$l_{v}^{f}$	$d_{v}^{b}$	$l_{v}^{b}$	*d_v_*	Δ*_v_*	*H_v_*	*W_v_*
A	3	0	1	3.7	3	3.7	0.10	0.88
B	3	0	1	3.7	3	3.7	0.10	0.88
C	3	1	2	2.7	6	1.7	0.20	2.33
D	6	0	1	3.7	6	3.7	0.25	2.155
E	6	1	1	2.7	6	1.7	0.25	2.155
F	3	1	2	2.7	6	1.7	0.15	2.305
G	3	0	1	3.7	3	3.7	0.05	0.855
H	3	0	1	3.7	3	3.7	0.10	0.88
I	3	0	1	2.7	3	2.7	0.15	1.005
J	3	1.8	4	1.7	12	0.1	0.55	5.065
K	3	2	3	1.7	9	0.3	0.45	3.795
L	2	3	3	1	6	2	0.35	2.375
M	1	3	3	0	3	3	0.10	0.95
N	1	2.8	4	0	4	2.8	0.15	1.395
O	1	4	3	0	3	4	0.15	0.875
P	1	4	3	0	3	4	0.20	0.9
Q	2	2.8	4	1	8	1.8	0.40	3.22
R	1	3.8	4	0	4	3.8	0.30	1.37
S	1	3.8	4	0	4	3.8	0.10	1.27

**Table 4.** Status of tables after storing trace records in Table 1. (**a**) TIME_TABLE in original schema; (**b**) TIME_TABLE_B and TIME_TABLE_F in new schema.

**START**	**END**	**LOC**	**START_TIME**	**END_TIME**

1	18	A	2	3
2	17	B	5	6
3	16	O	8	9
4	9	H	11	12
5	6	I	14	16
7	8	J	13	15
10	15	K	10	11
11	12	M	12	14
13	14	N	13	15
19	36	C	3	4
20	35	Q	5	7
21	34	O	8	9
22	27	K	10	11
23	24	M	12	14
25	26	N	13	15
28	33	H	11	12
29	30	I	14	16
31	32	J	13	15
37	46	C	1	2
38	45	Q	4	5
39	44	O	8	9
40	43	H	11	12
41	42	I	14	16
47	66	D	1	3
48	65	Q	5	7
49	64	O	8	9
50	55	H	11	12
51	52	I	14	16
53	54	J	13	15
56	63	K	10	11
57	58	M	12	14
59	60	N	13	15
61	62	N	14	16
67	82	F	3	6
68	81	O	8	9
69	74	H	11	12
70	71	I	14	16
72	73	J	13	15
75	80	K	10	11
76	77	M	12	14
78	79	N	13	15

(**a**) TIME_TABLE				

**START_B**	**END_B**	**LOC**	**START_TIME**	**END_TIME**

1	18	O	8	9
2	5	B	5	6
3	4	A	2	3
6	11	Q	5	7
7	8	C	3	4
9	10	D	1	3
12	15	Q	4	5
13	14	C	1	2
16	17	F	3	6

TIME_TABLE_B				

**START_F**	**END_F**	**LOC**	**START_TIME**	**END_TIME**

1	16	O	8	9
2	7	H	11	12
3	4	I	14	16
5	6	J	13	15
8	15	K	10	11
9	10	M	12	14
11	12	N	13	15
13	14	N	14	16

TIME_TABLE_F (**b**)				

**Table 5.** Representative query set.

**Query Number**	**Query**
Q1	<TAG_ID = my_tag_id>
Q2	<//A//B/C>
Q3	<//A//B[(EndTime-StartTime) < 50]/C>
Q4	<//A//B/C, Name = 'laptop'>
Q5	<COUNT(), //A//B/C>
Q6	<AVG(B.StartTime), //A//B/C>
Q7	<AVG(C.EndTime-B.StartTime), //A//B/C>
Q8	<MIN(B.StartTime), //A//B/C>
Q9	<MIN(C.EndTime-B.StartTime), //A//B/C>

**Table 6.** SQL statements of Q1.

<**TAG_ID** = **my_tag_id**>	
**SELECT**	P.ELEN, P.OEN
**FROM**	PATH_TABLE P, BUNDLE_TABLE B, TAG_TABLE T
**WHERE**	T.TAG_ID= ***my_tag_id*** AND B.BUNDLE_ID=T.BUNDLE_ID AND B.PATH_ID=P.PATH_ID

**Table 7.** SQL statements of Q2.

<//**A**//**B**/**C**>	
**SELECT**	T.TAG_ID
**FROM**	PATH_TABLE P, BUNDLE_TABLE B, TAG_TABLE T
**WHERE**	P.ELEN%(pA*pB*pC)=0 AND P.ELEN% ***pA***<P.ELEN% ***pB*** AND P.ELEN% ***pB***+1=P.ELEN%***pC*** AND B.PATH_ID=P.PATH_ID AND T.BUNDLE_ID=B.BUNDLE_ID

**Table 8.** SQL statements of Q3.

<//**A**//**B[(EndTime-StartTime)**<**50]**/**C**>	
**SELECT**	T.TAG_ID
**FROM**	PATH_TABLE P, BUNDLE_TABLE B, TAG_TABLE T, TIME_TABLE_B TB
**WHERE**	P.ELEN%(pA*pB*pC)=0 AND P.ELEN%***pA***<P.ELEN% ***pB*** AND P.ELEN% ***pB***+1=P.ELEN% ***pC*** AND B.PATH_ID=P.PATH_ID AND T.BUNDLE_ID=B.BUNDLE_ID AND TB.LOC='B' AND TB.START_B<=B.START_B AND TB.END_B>=B.END_B AND TB.END_TIME-TB.START_TIME<50
**UNION**	
**SELECT**	T.TAG_ID
**FROM**	PATH_TABLE P, BUNDLE_TABLE B, TAG_TABLE T, TIME_TABLE_B TF
**WHERE**	P.ELEN%(pA*pB*pC)=0 AND P.ELEN%***pA***<P.ELEN% ***pB*** AND P.ELEN%pB+1=P.ELEN% pC AND B.PATH_ID=P.PATH_ID AND T.BUNDLE_ID=B.BUNDLE_ID AND TF.LOC='B' AND TF.START_B<=B.START_B AND TF.END_B>=B.END_B AND TF.END_TIME-TF.START_TIME<50
